# Supplementary material for: Physiological and Transcriptome Analysis on Diploid and Polyploid Populus ussuriensis Kom. under Salt Stress
Source: Int J Mol Sci. 2022 Jul 7;23(14):7529. doi: 10.3390/ijms23147529 (PMC9319462; doi:10.3390/ijms23147529)
Supplement: Supplementary file 1 [file ijms-23-07529-s001.zip › Supplementary Figures.pdf]

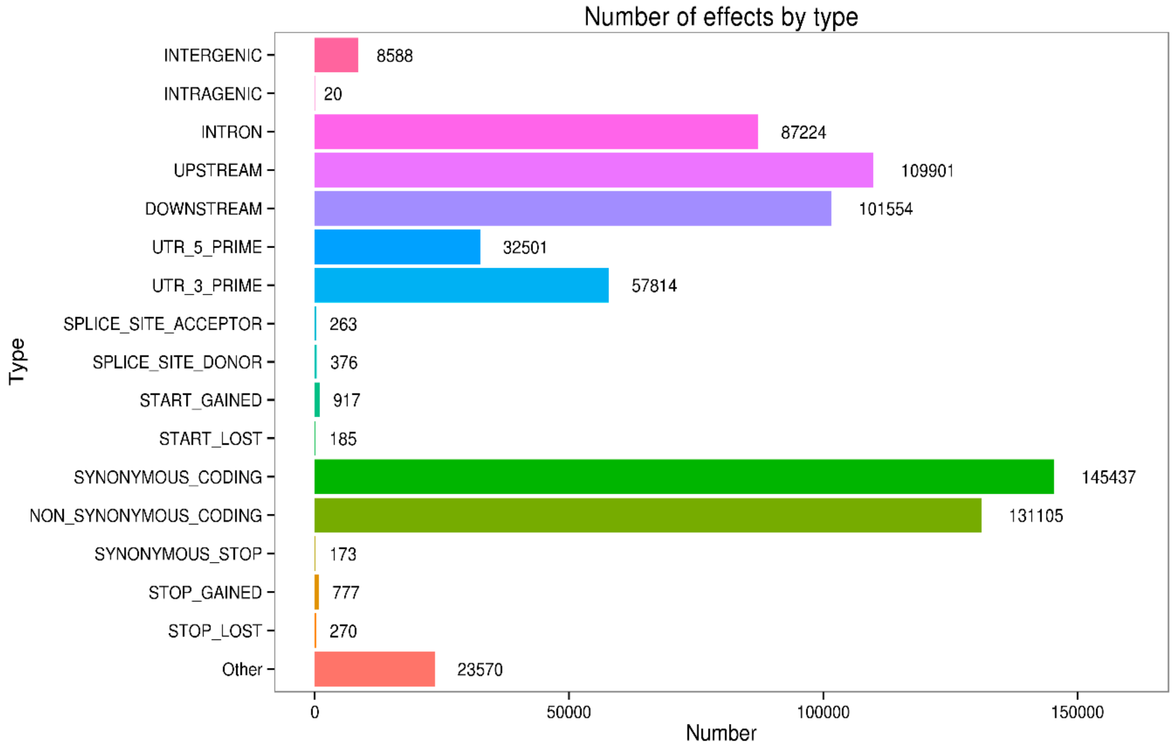

Figure S1 The classification and annotation of all the SNPs in CK, T12 and F20 samples before and after salt stress.

a

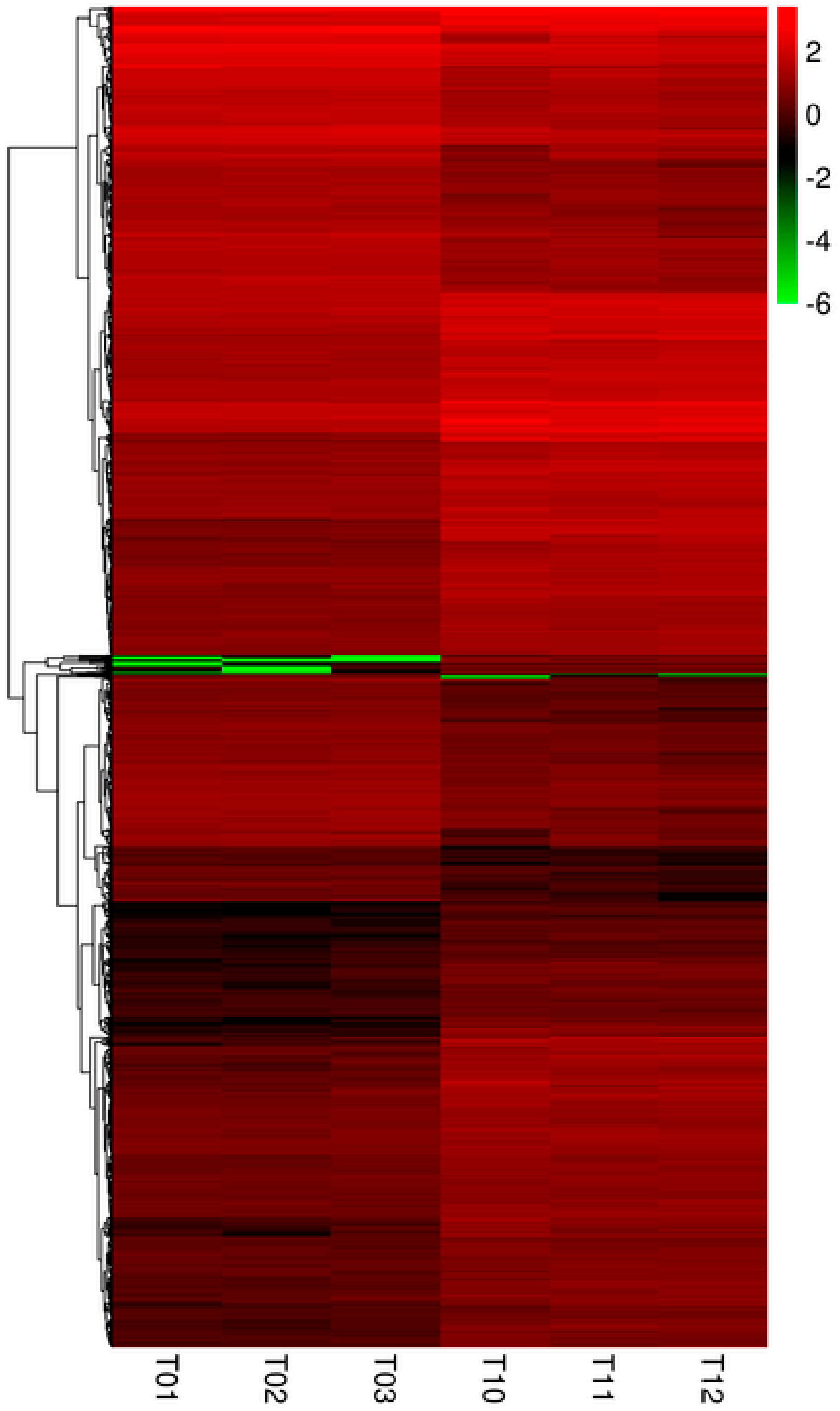

b

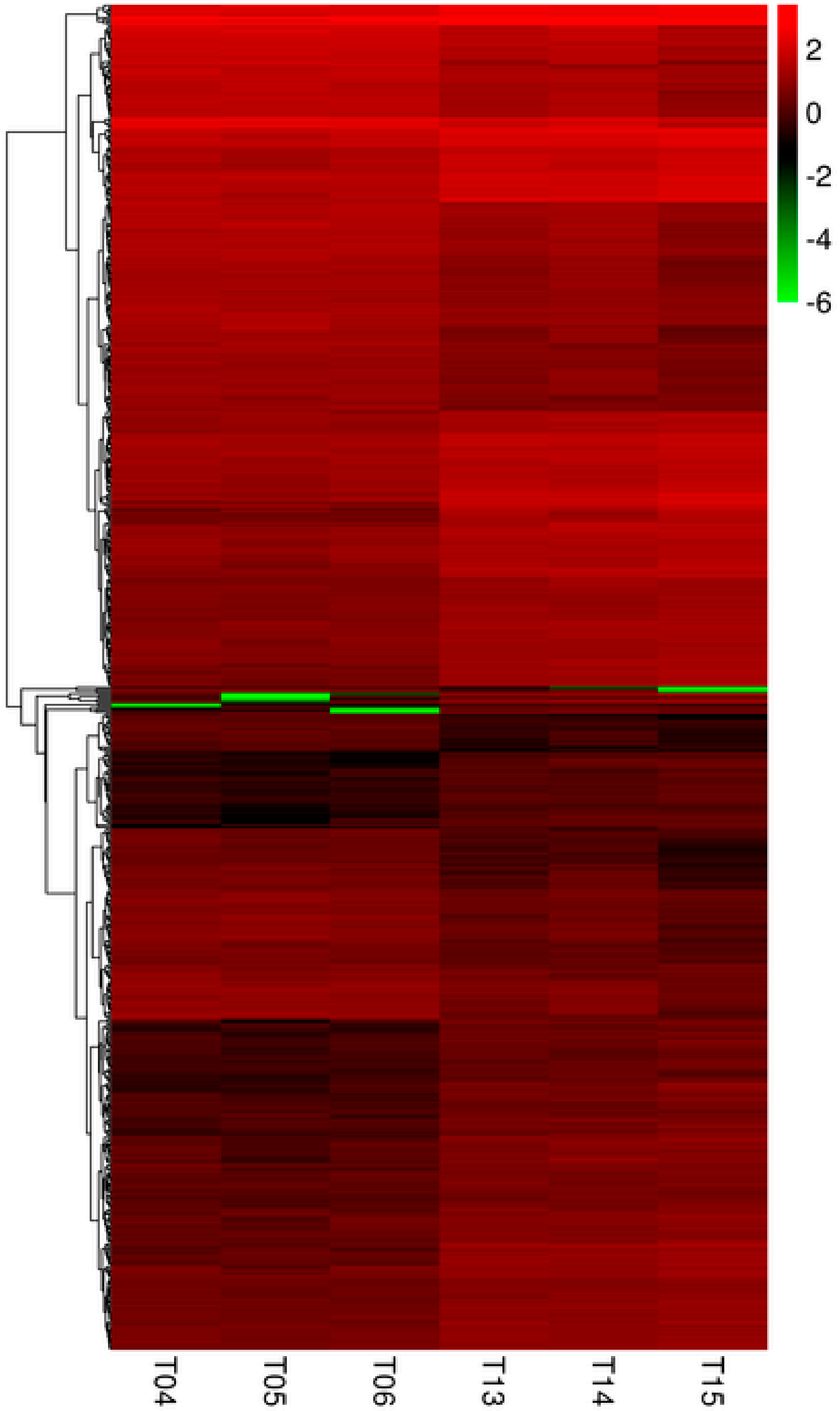

c

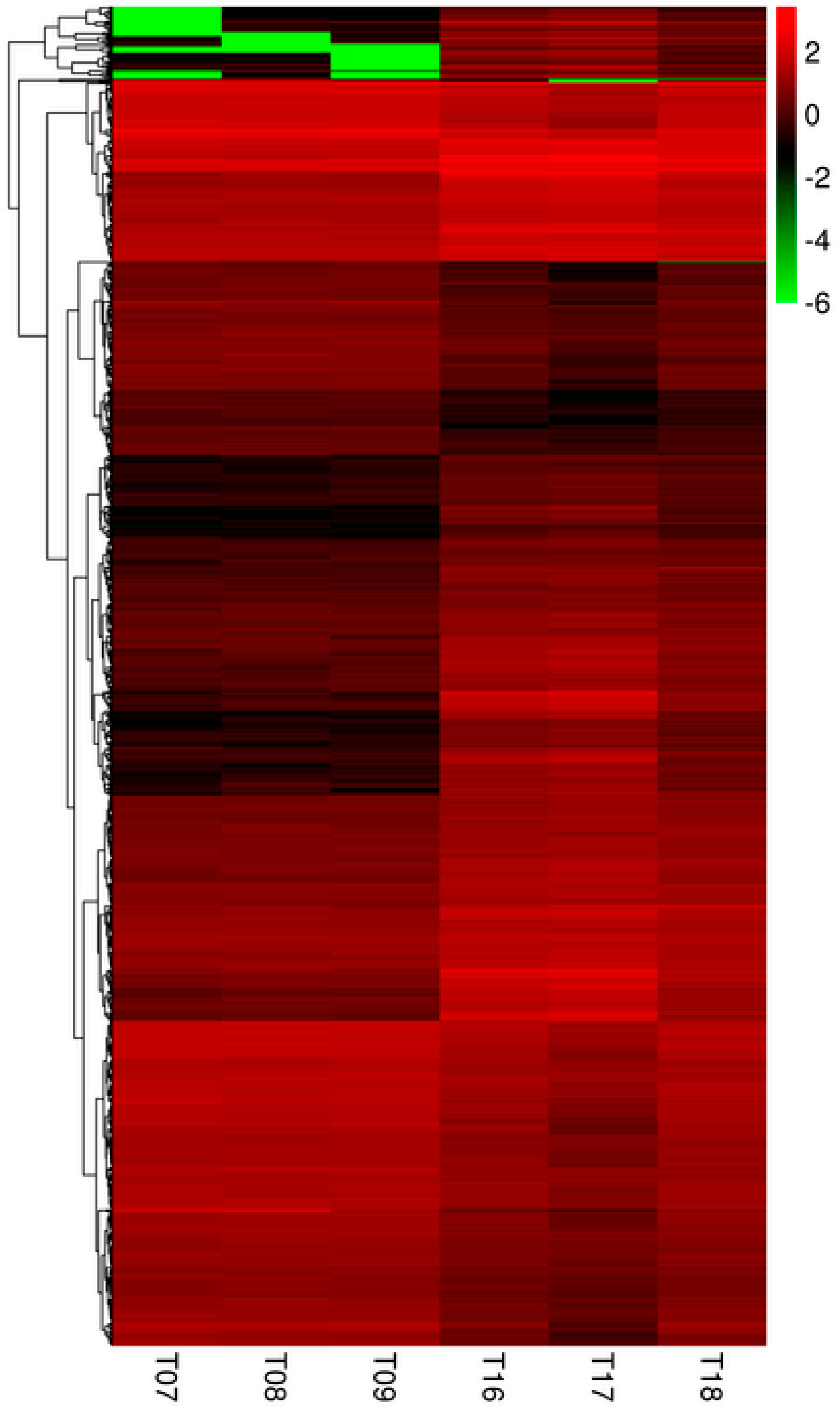

Figure S2 Cluster analysis of the DEGs of CK, T12 and F20 samples before and after salt stress. a. T01, T02, T03 indicate three biological repetitions of CK samples before salt stress, while T10, T11, T12 indicate three biological repetitions of CK samples 36h after salt stress; T04, T05, T06 indicate three biological repetitions of T12 samples before salt stress, while T13, T14, T15 indicate three biological repetitions of T12 samples 36h after salt stress; T07, T08, T09 indicate three biological repetitions of F20 samples before salt stress, while T16, T17, T18 indicate three biological repetitions of F20 samples 36h after salt stress.

a

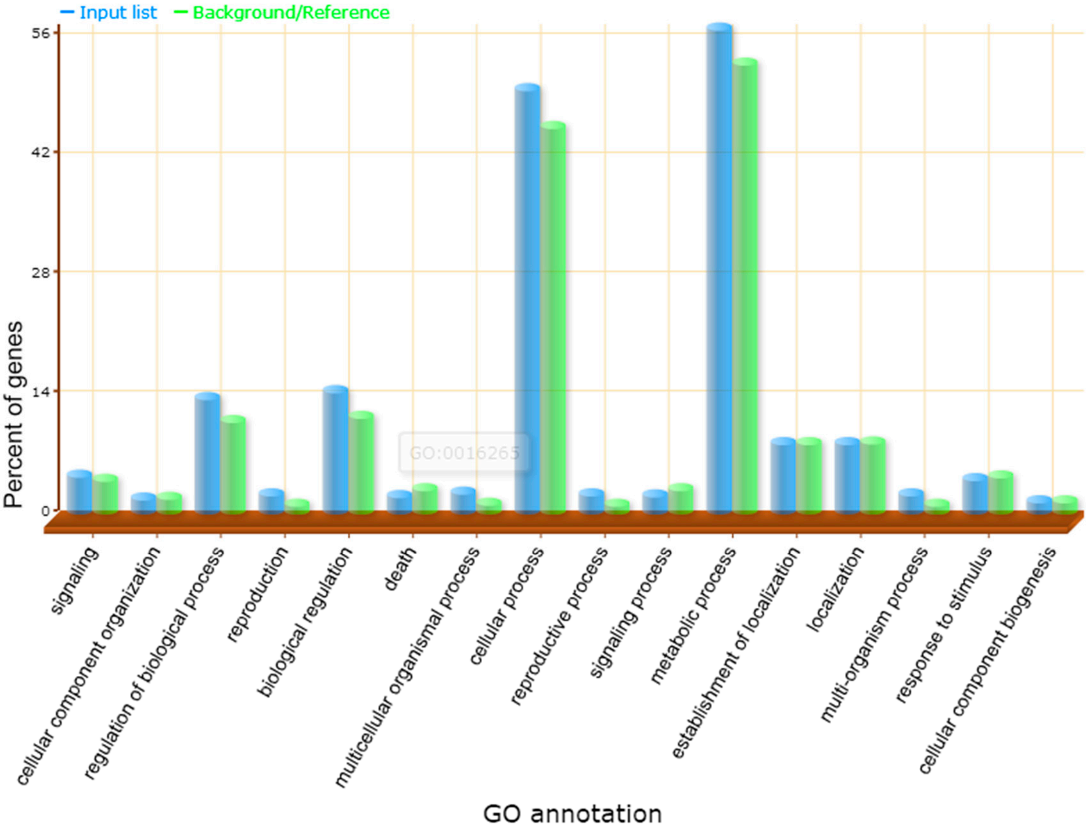

Input list
Background/Reference
56
42
28
14
0
Percent of genes
signaling
cellular component organization
regulation of biological process
reproduction
biological regulation
death
multicellular organismal process
cellular process
reproductive process
signaling process
metabolic process
establishment of localization
localization
multi-organism process
response to stimulus
cellular component biogenesis
GO annotation

b

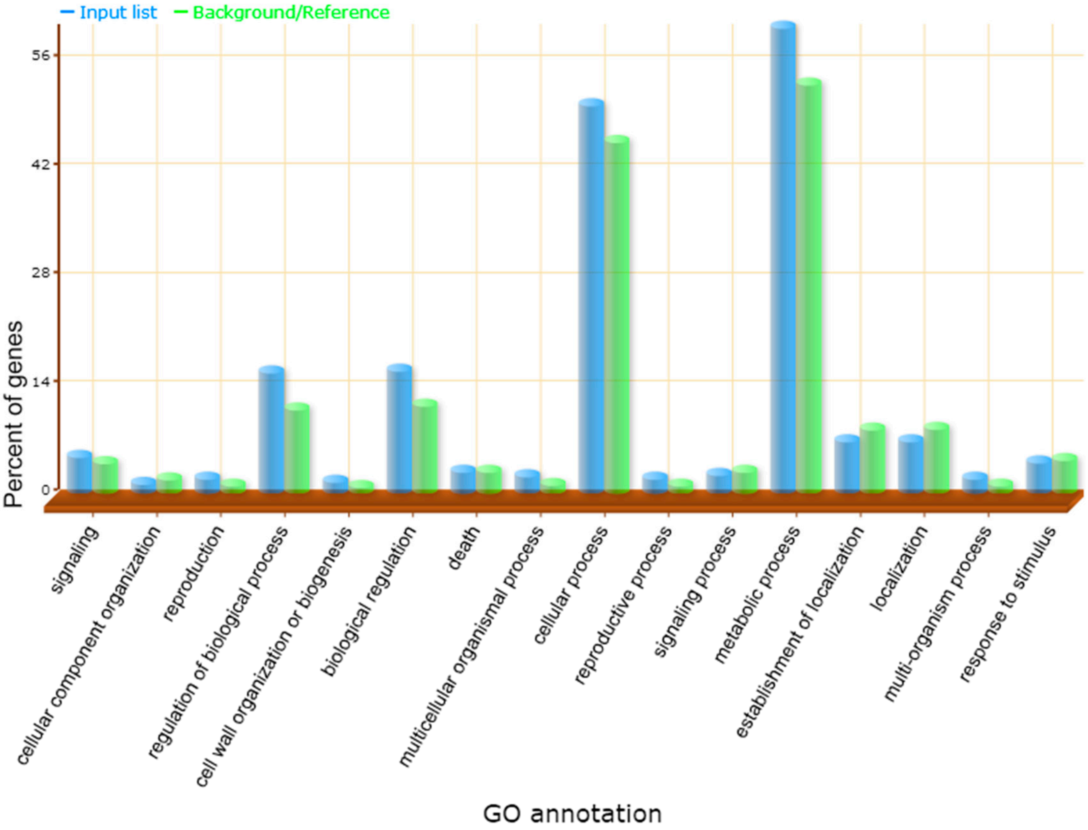

Input list
Background/Reference
56
42
28
14
0
Percent of genes
signaling
cellular component organization
reproduction
regulation of biological process
cell wall organization or biogenesis
biological regulation
death
multicellular organismal process
cellular process
reproductive process
signaling process
metabolic process
establishment of localization
localization
multi-organism process
response to stimulus
GO annotation

c

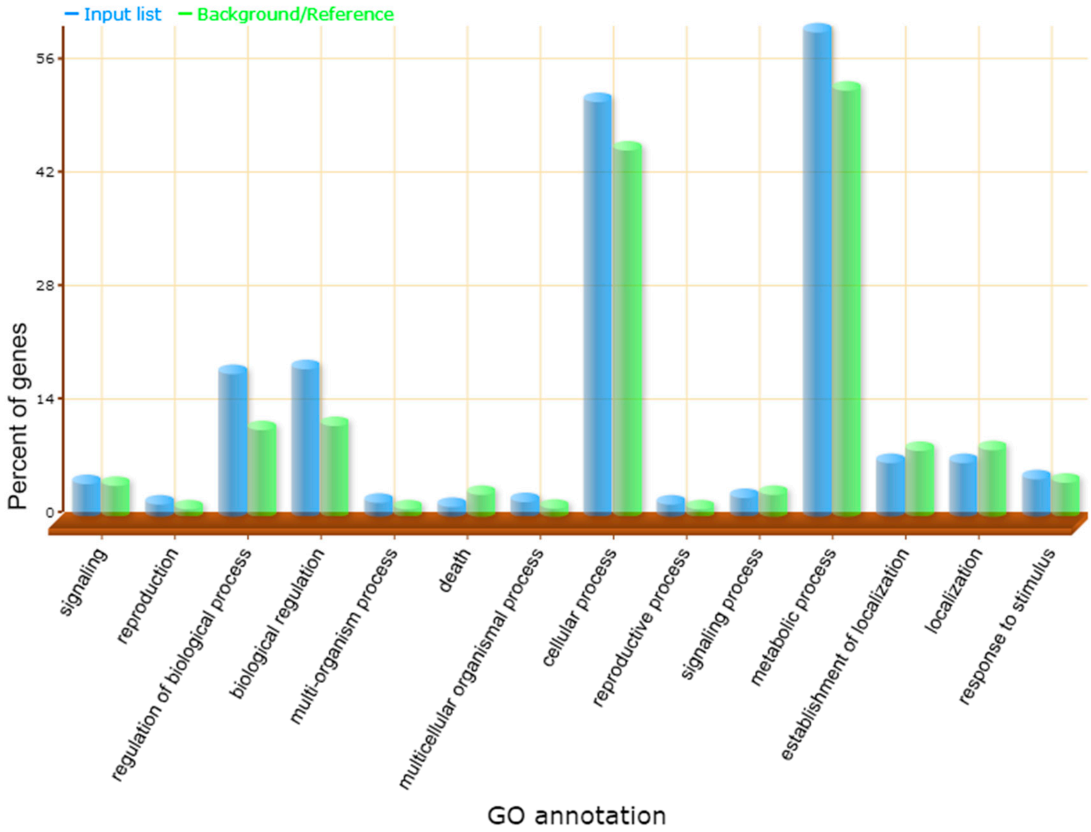

Figure S3 GO term enrichment on the DEGs of CK, T12 and F20 samples before and after salt stress. a. GO term enrichment on the DEGs between T01, T02, T03 and T10, T11, T12. **b.** GO term enrichment on the DEGs between T04, T05, T06 and T13, T14, T15. **c.** GO term enrichment on the DEGs between T07, T08, T09 and T16, T17, T18.
