# Supplementary material for: Physiological and Transcriptome Analysis on Diploid and Polyploid Populus ussuriensis Kom. under Salt Stress
Source: Int J Mol Sci. 2022 Jul 7;23(14):7529. doi: 10.3390/ijms23147529 (PMC9319462; doi:10.3390/ijms23147529)
Supplement: Supplementary file 1 [file ijms-23-07529-s001.zip › Table S1.pdf]

Table S1 The primers and reference genes that used in qRT-PCR validation.

Gene	Gene Classification	Forward primer (5′ – 3′)	Reverse primer (5′ – 3′)	Amplicon	
				L(bp)	Tm(°C)
Potri.001G044500	WRKY40	CTCGGTTCAGTACCCTGCTC	GGCCATCTGTTCCACCAAGA	156	60.0
Potri.005G069500	NAM	GAGGGTCAACAATGGGAGCA	TGGTGTTGAATCGGACGTGT	83	60.0
Potri.018G038100	AP2_ERF	CGGCGACGTTTCATTTTCCA	GCAACCTCCCTGATCTGCAT	88	60.0
Potri.008G091900	WRKY4	ATACACCAGCAGGCACTAGC	TGTGTCGAAGACATTGCCGA	104	60.0
Potri.005G001600	MYB	TCGGTCACTCGAAAACGACA	GCTTGTTGATGCTGGTTCGG	186	60.0
Potri.007G135300	NAC	CGGAGTGAAGTTTGACCCGA	GCCCATCTTTGCTCACTCCT	176	60.0
At4g33380-like	Housekeeping	CTGCCTCTGCTGATACCT	GGAACCGAACCAATCTTCTC	272	60.0

L, length of PCR production (in base pairs); Tm, melting temperature (in °C).
